# Imaging in Cancer Diagnosis: Consolidating Progress and Charting the Next Steps

**DOI:** 10.3390/tomography12100141

**Published:** 2026-09-28

**Authors:** Giovanni Sussan, Filippo Crimì

**Affiliations:** Radiology Institute, Department of Medicine-DIMED, University of Padova, 35128 Padova, Italy

## 1. Introduction

When this Special Issue opened in January 2024, its premise was straightforward but ambitious: imaging had moved well beyond a gatekeeping role ahead of biopsy, and the structural, functional, and metabolic data extracted from ultrasound, CT, MRI, and PET were becoming primary evidence in their own right, with artificial intelligence poised to expand what could be read from them [1,2]. Two and a half years and fifteen original contributions later (Table 1), it is a good moment to ask not only what this collection reported, but what it tells us about where oncologic imaging research is actually heading, and where the gaps identified at the outset remain open.

## 2. Overview of the Contributions

The most striking feature of the final collection is its center of gravity: half of the fifteen research articles focus on breast imaging, and nearly all of them share the same underlying strategy, pairing an established or emerging modality with a computational layer designed to extract information beyond what a reader can see directly. Radiomics and deep-learning models converted dynamic contrast-enhanced MRI kinetics and multimodal ultrasound data into predictors of molecular subtype, HER2 status, and lesion malignancy (Table 1, Nos. 7, 12 and 16), and several of these efforts went further, validating their output directly against a hard clinical endpoint, pathology or T-stage, rather than against another imaging read (Table 1, Nos. 4 and 8).

The remaining contributions apply this same underlying logic, converting an imaging signal into a reproducible, outcome-anchored biomarker, to other organ systems and clinical questions. In the thorax, a large retrospective series quantified how closely CT tumor sizing tracks pathology, isolating ground-glass opacity as the main source of disagreement relevant to staging (Table 1, No. 14). CT-derived liver volume was linked to long-term survival after colonic cancer resection (Table 1, No. 2), and delta-radiomics applied to serial imaging predicted chemotherapy response in liver metastases, arguably the collection’s clearest example of imaging guiding treatment rather than only diagnosis (Table 1, No. 6). Further contributions extended the same quantitative approach to thyroid and skin lesions (Table 1, Nos. 5 and 10).

**Table 1:** Contributions to the Special Issue “Imaging in Cancer Diagnosis”.

No.	Title	Authors
1	Introduction to Special Issue Imaging in Cancer Diagnosis (Editorial)	Zanon, C.; Quaia, E.; Crimì, F.
2	The Relationship between Liver Volume, Clinicopathological Characteristics and Survival in Patients Undergoing Resection with Curative Intent for Non-Metastatic Colonic Cancer	McGovern, J.; et al.
3	Comprehensive CT Imaging Analysis of Primary Colorectal Squamous Cell Carcinoma: A Retrospective Study	Yoon, E.J.; et al.
4	Digital Breast Tomosynthesis for Upgraded BI-RADS Scoring towards the True Pathology of Lesions Detected by Contrast-Enhanced Mammography	Grubstein, A.; et al.
5	Understanding the Dermoscopic Patterns of Basal Cell Carcinoma Using Line-Field Confocal Optical Coherence Tomography	Barbarossa, L.; et al.
6	Delta Radiomics and Tumor Size: A New Predictive Radiomics Model for Chemotherapy Response in Liver Metastases from Breast and Colorectal Cancer	Gennaro, N.; et al.
7	Distinguishing Low Expression Levels of HER2 in Breast Cancer: Insights from Qualitative and Quantitative MRI Analysis	Shen, Y.; Zhang, X.; et al.
8	AI-CAD-Guided Mammographic Assessment of Tumor Size and T Stage: Concordance with MRI for Clinical Staging in Breast Cancer Patients Considered for NAC	Park, G.E.; et al.
9	Quantitative Volumetric Analysis Using 3D Ultrasound Tomography for Breast Mass Characterization	Anzola, M.L.; et al.
10	Quantitative Ultrasound Grayscale Analysis and Size of Benign and Malignant Solid Thyroid Nodules	Sultan, S.R.; et al.
11	Clinically Focused Computer-Aided Diagnosis for Breast Cancer Using SE and CBAM with Multi-Head Attention	Ogut, Z.; Karaduman, M.; Yildirim, M.
12	Dynamic Contrast-Enhanced MRI Kinetic Curve-Driven Parametric Radiomics for Predicting Breast Cancer Molecular Subtypes: A Multicenter and Interpretable Study	Wang, T.; Gong, J.; et al.
13	Direct Segmentation of Mammography and Tomosynthesis Sinograms for Lesion Localization	Ruíz Muñoz, E.; et al.
14	Computed Tomography Versus Pathologic Tumor Size in Resected Lung Tumors: High Correlation, Limited Agreement, and the Impact of Ground-Glass Opacity	Yavuz, O.; et al.
15	Biophysical Diffusion MRI Models Better Identify White Matter Tracts in Edema	Prentiss, I.E.; Hakhu, S.; et al.
16	An Efficient Cross-Modal Interaction and Dynamic Fusion Network for Multimodal Breast Ultrasound Diagnosis	Wu, X.; Lan, Y.; Han, L.; Wang, P.

## 3. Progress Toward the Special Issue’s Founding Objectives

Viewed against the Special Issue’s original aim, to reduce reliance on invasive procedures in patients with a low likelihood of malignancy through the use of imaging, the findings are genuinely encouraging. Four contributions (Table 1, Nos. 2, 4, 8 and 14) validate non-invasive imaging biomarkers directly against pathology or long-term outcome rather than against another imaging surrogate. That is the same evidentiary standard that, in a landmark prostate MRI trial, was used to justify replacing systematic biopsy with imaging-targeted sampling [3], and reaching it in four independent studies within a single collection is not a small achievement. The AI and radiomics contributions in this collection (Table 1, Nos. 6, 7, 9, 11, 12, 13 and 16) go a step further: they show this validation increasingly happening at the pixel and feature level rather than only at the level of radiologist interpretation, exactly the expansion of tissue characterization that the founding editorial anticipated artificial intelligence would bring.

## 4. Critical Research Gaps

The collection is nonetheless narrower than the original call for papers. Positron emission tomography [4] and photon-counting computed tomography, both named explicitly in the opening editorial as technologies of interest and increasingly central to oncologic imaging more broadly, are not represented in any contribution, and prostate cancer, flagged there as a leading application of contrast-enhanced and elastographic ultrasound, is absent entirely, even though imaging-based risk stratification has already replaced systematic biopsy in that organ [3]. Methodologically, all but one study (Table 1, No. 12) is single- or dual-center and retrospective, sample sizes for the AI models rarely exceed a few hundred patients, and none report the external, prospective, multi-vendor validation that current methodologic guidance for diagnostic AI considers a prerequisite for clinical claims [5]. None of the deep-learning contributions (Table 1, Nos. 11, 13 and 16) report an interpretability analysis, an omission that matters increasingly for the regulatory and clinical acceptance of image-derived biomarkers [6].

## 5. Future Research Priorities

Building on both what this Special Issue achieved and what it left open, several priorities stand out for the next phase of research in this field.

The clearest priority is that external validation must become the norm rather than the exception. Current methodologic guidance for diagnostic AI already treats prospective, multicenter testing on pre-specified, externally sourced data as a prerequisite for any clinical claim [5], and this collection’s own multicenter study (Table 1, No. 12) shows that such a design is achievable within a special issue’s typical scope, even if it remains the exception rather than the rule: audits of published imaging-AI studies continue to find that most still report performance from small, single-institution, retrospective cohorts [7]. Reporting frameworks such as CLAIM [8] give authors and reviewers a concrete checklist for closing that gap, and public repositories such as The Cancer Imaging Archive already offer a route to the external, multi-vendor test sets that validation requires [9].

Equally important, the field needs to confront where its training data comes from: deep-learning cohorts have been shown to cluster around a small number of academic centers [10], and federated learning—training models across institutions without centralizing patient data—offers one practical way to broaden that base while respecting data-governance constraints [11].

Explainability and measurement standardization, in turn, are two sides of the same reproducibility problem raised by this issue’s own deep-learning contributions in Section 4. Radiologists and regulators need to see which image regions and features drive a given prediction [6], and the radiomic and deep-learning features feeding those predictions need to be computed consistently across software and institutions, which is precisely the aim of ongoing efforts such as the Image Biomarker Standardization Initiative [12].

Beyond methodology, broader modality and disease coverage remains the most direct way to close the gap identified above: extending this body of work to PET and other functional or metabolic imaging, photon-counting CT, and prostate and other genitourinary cancers that were part of the original scope but did not materialize in submissions, with prostate MRI’s own standardized reporting system offering a template for how a new modality moves from research finding to structured clinical use [13].

A further, and perhaps the most promising, direction is extending imaging biomarkers from diagnosis into treatment monitoring and surgical planning, building directly on the delta-radiomics and diffusion-tractography contributions already in this issue (Table 1, Nos. 6 and 15), and the broader move toward integrating imaging with genomic and clinical data already under way in breast oncology [14]. As models trained on imaging, text, and other clinical data mature, foundation models may eventually offer a shared backbone for many of these tasks rather than a bespoke network for each one [15].

Finally, none of this will reach patients without a credible regulatory and economic case. Authorization pathways for AI-based devices are still evolving unevenly across major jurisdictions [16], and evidence on whether these tools are worth their cost, rather than simply accurate, remains thin [17]. Building that evidence base should be part of the research agenda, not an afterthought to it.

## 6. Conclusions

The founding editorial of this Special Issue envisioned imaging capable not only of describing a tumor but of substituting, in low-risk patients, for the biopsy it aims to spare, provided radiologists develop the subspecialized expertise and artificial intelligence reaches the maturity such a role demands. Having followed this Special Issue from its first submission to its last, we find that vision still holds, and perhaps more urgently than when it was written: what remains is to make it operational, embedding validated, quantitative imaging biomarkers into standardized reporting frameworks of the kind referring physicians already trust [18], training the subspecialized readers this field will increasingly require as its techniques multiply, and holding artificial intelligence models to the same evidentiary bar expected of any other diagnostic test before they enter routine oncologic care [19]. As radiologists working daily alongside oncologists and surgeons, we know that the real measure of this progress will not be a reported accuracy figure, but how many patients are spared an unnecessary biopsy and how many cancers are caught early enough to change their course. We hope this collection, and the questions it leaves open, continue to move the field a little closer to that outcome.
